# The Impact of Financial and Macroeconomic Shocks on the Entropy of Financial Markets

**DOI:** 10.3390/e21030316

**Published:** 2019-03-23

**Authors:** Sorin Anagnoste, Petre Caraiani

**Affiliations:** 1 Faculty of Business Administration in Foreign Languages, Bucharest University of Economic Studies, 010374 Bucharest, Romania; 2 Institute for Economic Forecasting, Romanian Academy, 050711 Bucharest, Romania

**Keywords:** entropy, financial markets, monetary policy, networks

## Abstract

We propose here a method to analyze whether financial and macroeconomic shocks influence the entropy of financial networks. We derive a measure of entropy using the correlation matrix of the stock market components of the DOW Jones Industrial Average (DJIA) index. Using VAR models in different specifications, we show that shocks in production or the DJIA index lead to an increase in the entropy of the financial markets.

## 1. Introduction

The use of networks to analyze economic and financial phenomena is developing fast. There have been numerous recent significant contributions that have further developed our understanding about the potential of using networks or network-based perspectives in studying financial phenomena (see [1,2,3,4,5,6,7,8,9]).

Among the approaches used, the entropy-based methods have provided valuable results. We can include here the contribution of [10] who approached the relationship between the largest 187 financial companies in the world based on transfer entropy. His study showed a central role for insurance companies from the largest two economies, that of the United States and that of the Euro Area.

Another strand of research using entropy has focused on its capacity to reveal the state of the financial markets. For example, Ref. [11] has shown that we can derive a time-varying entropy using the singular value decomposition of the matrix of correlations of stock markets and that, furthermore, this entropy has predictive power with respect to the stock market dynamics. The results here have been replicated in [12] for the Shenzhen stock market for the use of entropy within a multiscale framework.

A related research looked at the long run relationship between stock and commodities (see [13]). The main result was that there is a decoupling between commodities and equity markets. Another use of entropy has been done by [14], who used the permutation entropy to look at whether the complexity or degree of information varies during market crashes. The main result, mirroring earlier contributions, was the permutation entropy decreases during market crashes.

In a recent paper, Ref. [15] has shown that we can also use entropy derived on the basis of correlation matrices to study the transmission of entropy shocks among different markets (which can be understood as a sort of informational shocks between the different financial markets).

In this paper, we aim at building upon recent contributions on the use of entropy in modelling financial markets. Specifically, we aim at looking at whether and how macroeconomic and financial shocks contributed to the variation in entropy of financial markets. To achieve this, we built on previous studies that also used time-varying measures of entropy derived from correlation matrices (see [11] or [15]), and propose the use of structural series approach that allows us to study the impact of macroeconomic and financial shocks on the entropy of financial markets.

There are several contributions that we make in this paper. First, we propose one of the first studies that approaches the changes of the structure of networks following macroeconomic and financial shocks. Second, we characterize the changes in the networks through the use of entropy derived on the basis of matrix correlations of stocks composing the network.

## 2. Methodology

This section details the methods and tools used in the paper. The techniques are quite standard; however, the presentation might be of interest to those not familiar with it.

### 2.1. Correlation Networks of Stocks

Our approach to construct correlation-based matrices is standard. We look at the correlation between the stock components of the DOW Jones Industrial Average index (that includes 30 stocks, see Appendix A). There are many ways to construct networks of financial stocks, from correlations (see [16]), to variance decompositions using VAR models (see [17]).

We use here the simple correlation between two stocks, which is given by:

(1) $$\rho_{i,j}=\frac{cov(r_{i},r_{j})}{\sigma_{r_{i}}\sigma_{r_{j}}}.$$

$r_{i}$ stands for the return of a stock *i*. We denote by $\sigma_{r_{i}}$ the standard deviation of the return of stock *i*. The return of a stock *i* is given by the logarithmic difference:

(2) $$r_{i}\left(t\right)=log\left[P_{i}\left(t\right)\right]-log\left[P_{i}\left(t-1\right)\right].$$

Here, $r_{i}\left(t\right)$ stands for the return of the stock *i* at *t*. By $P_{i}\left(t\right)$, we denote the value of the stock *i* in *t*.

Using the 30 stocks of the DJIA index, we derive a matrix of correlations which stands for the adjacency matrix which can be further used to derive financial networks. At the same time, although the correlation matrix can be used to represent a financial network, we rather focus on the (time-varying) properties of the correlation matrices, as seen from the next sections.

### 2.2. Singular Value Decomposition

The singular value decomposition (SVD, hereafter), when applied to a matrix allows us to obtain the following decomposition:

(3) $$A=USV^{T}.$$

Here, we applied the decomposition to a given matrix *A*, with *m* rows and *n* columns. The matrix *U* has *m* rows and *k* columns, while *V* has *n* rows and *k* columns. The matrix *S* can then be written by:

(4) $$S=\mathrm{diag}(\lambda_{1},\lambda_{2},\ldots ,\lambda_{k}),$$

where *k* is determined through the expression k=min(m,n). The derived matrix *S* has two important properties, namely positive elements as well as a decreasing order for the values of $\lambda_{k}$.

It must be underscored (we thank a reviewer for pointing this out) that the initial matrix is squared and we can just define the $\lambda_{t}$ as the eigenvalues of the correlation matrix.

### 2.3. Singular Value Decomposition-Based Entropy

We use the SVD approach to perform a decomposition of the correlation matrix and compute the entropy associated to the correlation matrices. We call this entropy the singular value decomposition-based entropy, denoted by *E*, throughout the text. The construction of the *E* measure of entropy is based on previous contributions by [18] who built on the reference contribution by [19].

The entropy of a correlation matrix can be derived in a simple and intuitive manner. We use the singular values obtained above and denoted by $\lambda_{k}$ in order to compute the normalized values denoted by ${\bar{\lambda }}_{k}$:

(5) $${\bar{\lambda }}_{k}=\frac{\lambda_{k}}{\sum \lambda_{k}}.$$

We use this relation to normalize the singular values obtained using the SVD approach. Next, we use the standard formula for entropy adapted to the use of singular values:

(6) $$E=-\sum {\bar{\lambda }}_{k}ln\left({\bar{\lambda }}_{k}\right).$$

As mentioned above, the variable *E* denotes the singular value decomposition-based entropy. More recent contributions with respect to deriving entropy measures for networks are provided by [20], for example.

A pertinent question would be about the economic significance of this measure. As discussed and shown in previous contributions, the entropy computed based on the singular value decomposition of the correlation network can depict the state of the financial markets and has a predictive ability with respect to the aggregate returns (see [11], [15] or [12]). As far as we are aware, this indicator has its own appealing, since it is derived from the correlation matrices of stock corresponding to the financial networks that can be constructed for these stocks, and it is hard to think to an alternative in the literature.

Furthermore, as previous research indicated that the entropy of financial networks can be taken as an indicator of the state of financial markets, our analysis allows for investigating if and how key macroeconomic and financial variables do influence it. The question is essential given the high interest of knowing what drives the dynamics of the financial markets, a question largely unanswered until now. Our approach does not propose a definite answer, but it rather suggests that using financial networks and the entropy derived on the basis of these networks can be used as a tool to look at fundamental drivers of the state of the financial markets, as given by the way key macroeconomic and financial variables affect the entropy.

## 3. Data

We selected data for the stock components of the Dow Jones Industrial Average Index, DOW30 (see Appendix A). We selected monthly data, since, in the second part of the analysis, we use the derived entropy measures to construct VAR models including monthly macroeconomic and financial data. At the same time, previous research on measuring the entropy based on the singular-value based approach (see [11]) has shown that using monthly or daily data do not lead to qualitatively or quantitatively different results.

The macroeconomic data include monthly time series on industrial production, inflation (using the consumer price index), the interest rate, as well as the Dow Jones Industrial Average (DJIA) composite index. For robustness, and given the fact that the nominal interest rate has reached the zero lower bound, we also considered as an alternative a shadow policy rate due to [21] (The nominal interest rate in the United States and other developed economies has reached the zero lower bound in the aftermath of the crisis. This has called for a new measure of interest rate that would allow measuring the actual stance of monetary policy. Such a proposed measure is the shadow interest rate). The sample for the DOW Jones Industrial Average components is between July 1991 and January 2019. Since we use a sliding window to compute the dynamic measure of entropy, the other macroeconomic and financial variables are taken from August 1994 to January 2019.

## 4. Results

### 4.1. The Singular Value Decomposition Based Entropy

In this section, we discuss the practical implementation of the algorithm to derive the entropy that was described earlier. Section 2.3 describes how one can determine the entropy for a given matrix of correlations (constructed as shown in Section 2.1). To determine the time-varying entropy, we use a sliding window of two years (24 observations) and of three years (36 observations), respectively. We use the log-returns of the stocks as given by Equation (2).

The size of the sliding window is set having in mind two criteria: the necessity of having a reasonable number of observations that would allow computing the correlations, and, second, ensuring enough observations for the second step of the analysis that consists of estimating VAR models. The second window is used in order to have an alternative measure for robustness.

To obtain the time-series for entropy, we start from the beginning of the sample and compute each time the entropy for the two windows of two and three years, respectively. In a recursive manner, we move each time one observation to the right.

In Appendix B, we show the log-difference of the entropy measure for the two different windows used. A few observations can be made. First, varying the size of the window does not lead to significantly different results. Secondly, the two financial crises in the sample, i.e., the 2001 tech crash (following the so called “*dot-com bubble*”) and the financial crisis between 2007 and 2008 are marked both through a highlighted decreased in entropy. This is also true for 1998, largely corresponding to the crisis in the Asian markets as well as the Russian crisis. The findings here confirm previous results found in [14] or [11].

### 4.2. Do the Macroeconomic and Financial Variables Granger-Cause the Entropy?

In this section, we take a first step towards answering the main research question of the paper: how do macroeconomic and financial shocks affect the entropy of the financial markets? We test here for Granger causality between interest rate (as well as shadow interest rate), the inflation, industrial production index as well as DJIA, on one hand, and the entropy (computed either using a 2-year or a 3-year window), on the other hand.

The role of this step is to establish, in a bivariate setting, whether the selected macroeconomic and financial variables have predictive power with respect to the entropy of financial markets. While this test is limited through the simple use of a bivariate setting and the lack of a structural model, it is still an important step before using a VAR model as we do in the next section.

The results are shown below in Table 1, Table 2, Table 3, Table 4 and Table 5. We considered only the case of the log-differenced entropy (in the literature, at the same time, one uses the interest rate as it is, and it does not compute the first difference). The approach is justified by the presence of unit roots (This is a standard way to verify whether a certain series is stationary or not, i.e., whether the series is characterized by a trend or not, either in the form of a deterministic or linear trend, or in the form of a stochastic trend. The presence of a unit root, implying non-stationarity, would distort the results for the Granger causality test) in the entropy taken as an index (see Appendix C).

Table 1, Table 2, Table 3, Table 4 and Table 5 show the results of testing for Granger causality. The results indicate some evidence of Granger causality for both types of interest rates (see Table 1 and Table 2). The evidence is robust also to changing the size of the window used to compute the entropy.

For the case of the industrial production, the evidence is the strongest, with evidence of Granger causality at almost all the lags. In contrast, there is no evidence for Granger causality from inflation to entropy. Finally, for the case of the DJIA index, the evidence that supports the Granger causality is limited to just one lag.

The evidence presented here, although not enough per se, justifies considering a more extensive econometric framework in the next section.

### 4.3. Analyzing the Impact of Monetary Policy Shocks on Entropy

In this section, we aim at answering the main research question in this paper: if and how financial and macroeconomic shocks affect the entropy of financial markets. To answer it, we build a VAR model consisting of monthly frequency series. We use the following set of variables: $y={\left[\Delta ip_{t},\Delta cpi_{t},r_{t},\Delta E_{t},\Delta ret_{t}\right]}^{\prime }$, where $ip_{t}$ is the monthly index of industrial production, $cpi_{t}$ is the consumer price index, $r_{t}$ stands for the interest rate, $E_{t}$ is the entropy measure and, finally, $ret_{t}$ represents the index level of DJIA. All variables with a Δ in front are log-differenced.

Formally, the VAR model is written as follows:

(7) $$y_{t}=c+A_{1}y_{t_{1}}+\ldots +A_{p}y_{t-p}+u_{t}.$$

$y_{t}$ stands for the vector of time series as detailed above, having a dimension k×1, *c* is a vector of constants k×1, $A_{i}$ stand for matrices of coefficients of dimension k×k and $u_{t}$ stands for a vector of dimensions k×1 of innovations. We also assume that $u_{t}$ is white noise, i.e., $E(u_{t})=0$, $E(u_{t}u_{t}^{\prime })=\Sigma$ with Σ the covariance matrix and $E(u_{t}u_{s}^{\prime })=0$, for t≠s.

The variables, except the interest rate (as it is a custom in the literature), are log-differenced, since they have a unit root (see Appendix C). We tested for the lag order using multiple information criteria. For both models considered, the tests indicate that the optimal number of lags is of two, see Appendix D.

We consider a baseline estimation, with the federal funds rate as the measure for the interest rate, and, additionally, the shadow policy rate, for an alternative estimation. Furthermore, for robustness, we also consider an alternative measure of entropy based on a three-year moving window.

To identify the structural shocks, including the monetary policy shocks, we use a Choleski ordering of the variables, with the following ordering: industrial production, consumer price index, interest rate, entropy, DOW 30. This is a rather typical ordering, with the variables moving the fastest, i.e., the stock market variables (including the entropy) ordered as last. The setting implies that the industrial production and the consumer price index do not contemporaneously react to interest rate changes, while the interest change does not respond to contemporaneous changes in the entropy measure or in the stock market.

This approach allows us to uncover whether the macroeconomic and financial variables considered here have any statistically significant impact on the main variable of interest, i.e., the entropy of the financial markets. The approach relies on a certain setting for identifying the structural shocks, see above, and thus it is better fit to answer the main question of the paper as compared to the simple Granger test for causality.

The results for the estimated baseline VAR models using the federal funds rate as the interest rate are shown below. We present the impact of the different structural shocks on entropy. The *x*-axis indicates the horizon of the response in months. log_entropy2y is the log-difference for entropy using a two-year window, while log_entropy3y is computed using a 3-year window, log_ip is the log-difference for industrial production, intrate is the interest rate used in the model and log_dow is the log-difference of the DJIA index.

**Figure 1:**
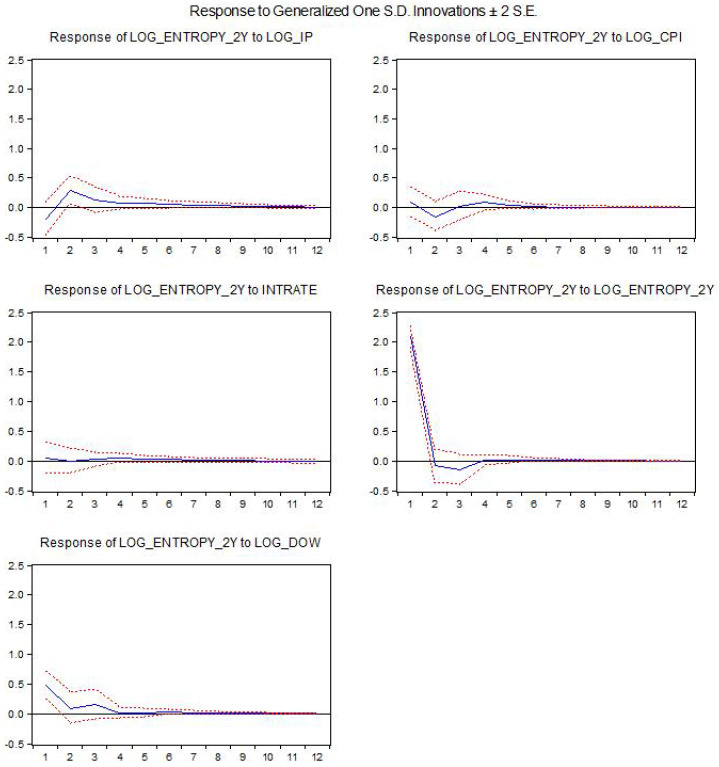
The impact of the structural shocks for the 2-year window entropy.

**Figure 2:**
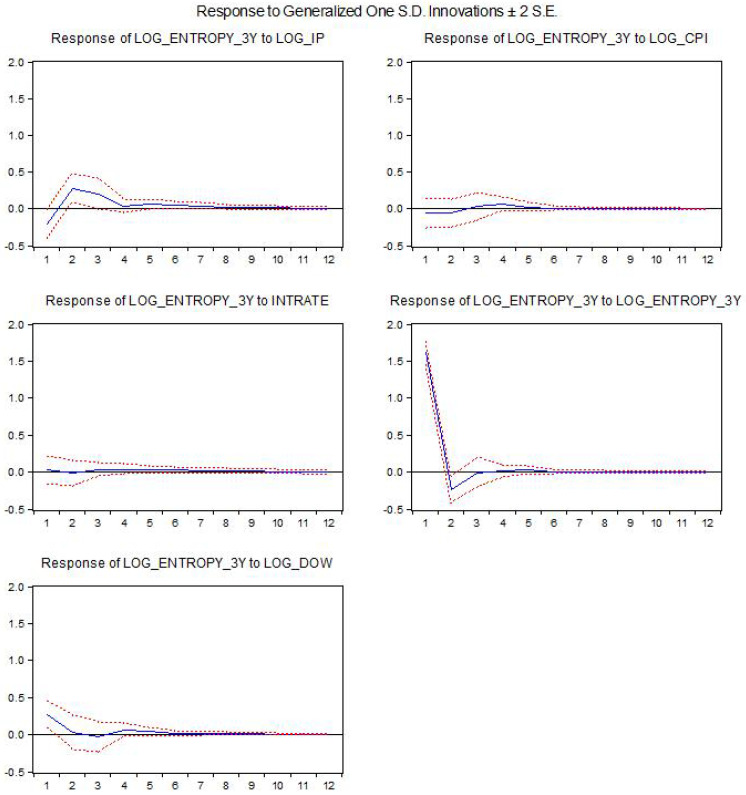
The impact of the structural shocks for the 3-year window entropy.

Figure 1 and Figure 2 show the impact of structural shocks from the baseline VAR model on entropy. We remark positive and statistically significant responses following shocks in the industrial production or the DJIA index. The results are robust to the use of a different window to compute the entropy. There is also a slightly positive response following a monetary policy shock; however, the response is not statistically significant (i.e., the confidence bands include the zero value).

We also performed a robustness exercise, using a similar setting and the same variables, except the interest rate, for which the shadow rate is used. The alternative estimates are shown in Appendix E. When looking at the results, we can see that the findings are similar to the ones discussed above. Again, there are positive responses following shocks in industrial production and DJIA.

This section provides a tentative answer to the main question of the paper: if and how do the macroeconomic and financial variables influence the entropy measure of financial markets. We found that there is a statistically significant role for industrial production and the DJIA index. While we would have expected a statistically significant impact of the stock market dynamics, the finding related to the industrial production index raises interesting issues that are worth studying further.

This finding could imply that the connections between the nodes of the financial market also reflect a strong connection with the real economy. On one hand, we know that stocks tend to organize themselves in financial networks in clusters following the specific of their activity (see, for example, [5]). On the other hand, the link could also potentially indicate that the correlations between the stocks become stronger or weaker following also the changes in the real economy.

## 5. Discussion

The main purpose of this study was to extend the previous literature regarding the modelling of the entropy within a macro-finance framework. We started by considering the derivation of entropy based on a moving window consisting of the DOW 30 index components for which we computed the correlations. Using a singular value decomposition, and a sliding window of 2 or 3 years of observations, we were able to derive a time-varying measure of entropy.

The principal contribution of the paper was to use the derived measure of entropy within a VAR framework including the industrial production, the inflation, the interest rate, the entropy as well as the DOW 30 returns. In this modelling framework, we were able to determine the impact of financial and macroeconomic shocks on entropy.

In our VAR framework, entropy responds positively to shocks in the monetary policy, although the impact is hardly statistically significant. However, there are positive responses that are statistically significant following industrial production shocks (after 2–3 months), as well as a positive response after a shock in the stock market (DJIA).

Furthermore, the results are robust to the use of different interest rate measures (the federal funds rate or the shadow policy rate), as well as to the different ways to construct the entropy (by varying the size of the moving window).

Compared to the results when using the simpler bivariate Granger causality tests, we see that the industrial production leads again to significant responses in entropy, while the inflation rate does not significantly impact it. There is a positive impact by the interest rate, although barely statistically significant. However, the DJIA index has a significant impact, although the results for the Granger causality tests were quite weak. The two frameworks are, however, not fully comparable since the VAR framework is multivariate and uses a certain procedure for identifying the structural shocks, while the Granger causality test is a simpler bivariate framework.

This is one of the first contributions not only to employ the entropy of a financial network in a typical macro-financial model, but also to obtain evidence regarding the movement of entropy following macroeconomic and financial shocks. The results are significant, since they show that the networks are not static at all, but they do react to changing in the macroeconomic and financial conditions.

The contributions here can be further developed and tested in many ways, and they show that there is a significant potential for entropy measures of networks to be used within standard macroeconomic and financial models.

## Figures and Tables

**Table 1:** Granger Causality Test: federal funds rate and entropy.

Lag	Entropy—2 years	Entropy—3 years
1 lag	0.08864	0.04869
2 lags	0.63769	0.82766
3 lags	0.36783	0.63527
4 lags	1.40247	1.29261
5 lags	2.14235 *	2.01708 *
6 lags	1.88353 *	1.88962 *
12 lags	1.36569	1.35294

Note: * denotes statistical significance of the F-test at 0.10 level.

**Table 2:** Granger Causality Test: shadow policy rate and entropy.

Lag	Entropy—2 years	Entropy—3 years
1 lag	0.36470	0.17885
2 lags	0.20665	0.41831
3 lags	0.32268	0.37718
4 lags	1.77009	0.90875
5 lags	3.09571 ***	2.26520 **
6 lags	2.68731 **	2.11641 *
12 lags	2.10438 **	1.56049

Note: * denotes statistical significance of the F-test at 0.10 level; ** statistical significance at 0.05 level and *** at 0.01 level.

**Table 3:** Granger Causality Test: industrial production and entropy.

Lag	Entropy—2 years	Entropy—3 years
1 lag	8.3364 ***	11.0945 ***
2 lags	4.69900 ***	8.38152 ***
3 lags	3.07081 **	6.47613 ***
4 lags	2.67924 **	4.19331 ***
5 lags	2.12966 *	3.38262 ***
6 lags	1.79666 *	3.03530 ***
12 lags	1.44856	2.41219 ***

Note: * denotes statistical significance of the F-test at 0.10 level; ** statistical significance at 0.05 level and *** at 0.01 level.

**Table 4:** Granger Causality Test: inflation rate and entropy.

Lag	Entropy—2 years	Entropy—3 years
1 lag	0.50872	0.01496
2 lags	0.63581	0.57440
3 lags	0.60068	0.87561
4 lags	0.76691	1.17148
5 lags	0.66059	0.94032
6 lags	0.81067	0.87214
12 lags	0.54968	0.97453

**Table 5:** Granger Causality Test: DJIA index and entropy.

Lag	Entropy—2 years	Entropy—3 years
1 lag	1.38714	1.66520
2 lags	2.45065 *	0.82142
3 lags	1.64917	0.92184
4 lags	1.48291	0.66261
5 lags	1.35815	1.24521
6 lags	1.14797	1.39846
12 lags	0.73223	1.01565

Note: * denotes statistical significance of the F-test at 0.10 level.

**Figure A1:**
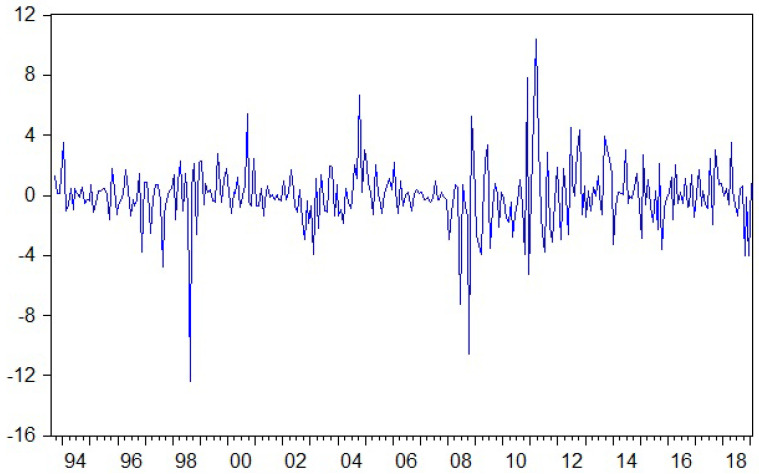
Singular Value Based Entropy using a 2-year window.

**Figure A2:**
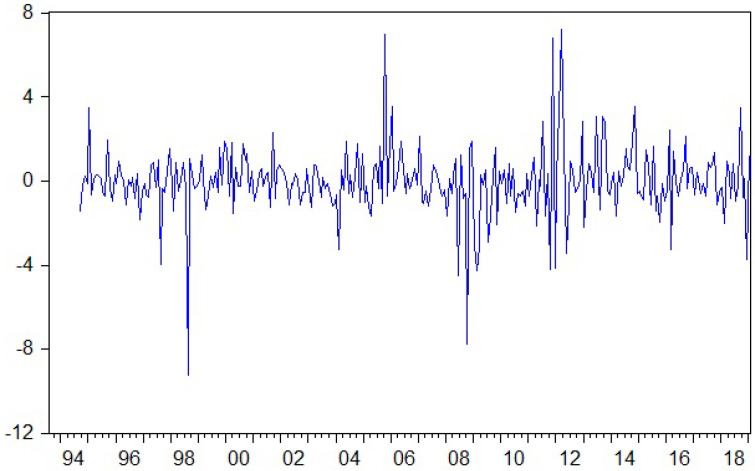
Singular Value Based Entropy using a 3-year window.

**Figure A3:**
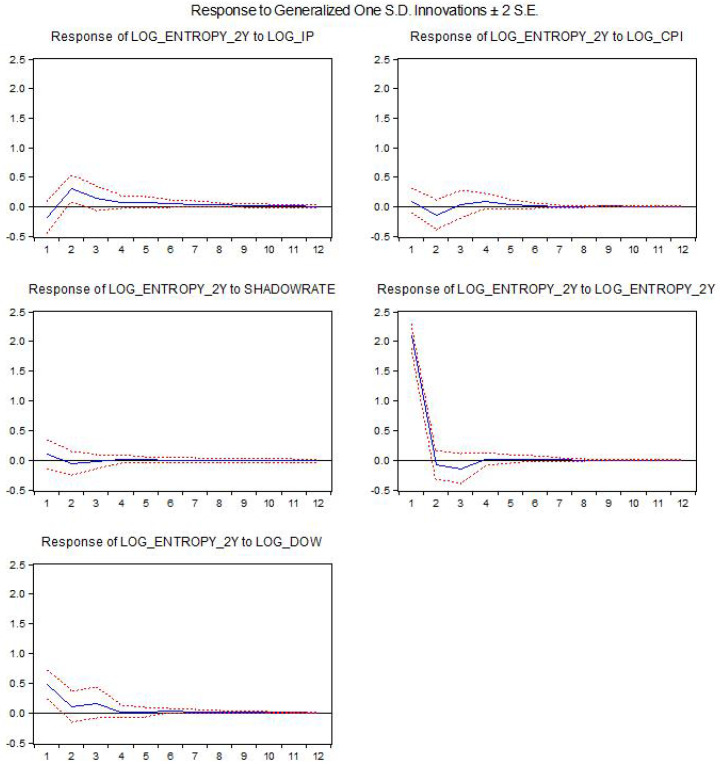
The impact of the structural shocks for the 2-year window entropy—robustness results based on the shadow interest rate.

**Figure A4:**
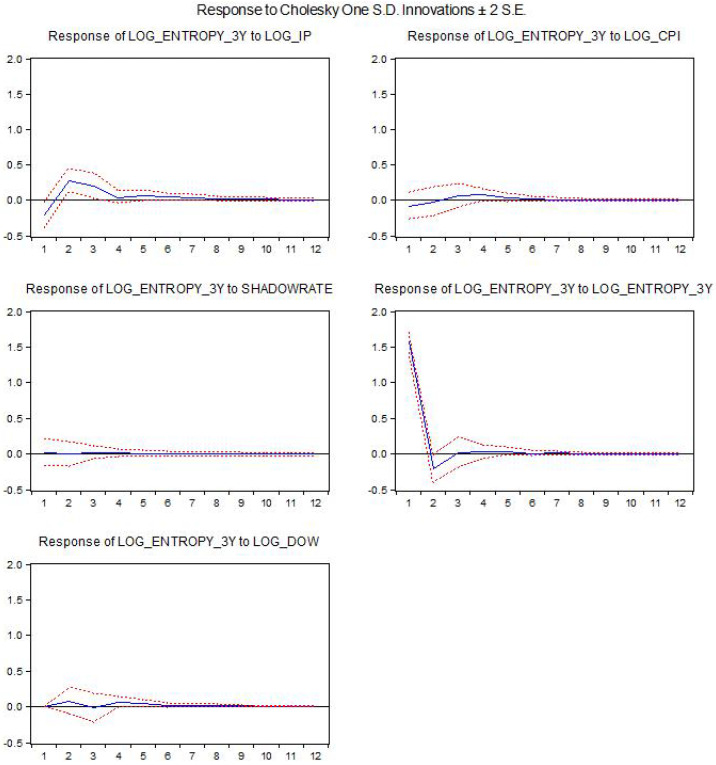
The impact of the structural shocks for the 3-year window entropy—robustness results based on the shadow interest rate.

## Abbreviations

The following abbreviations are used in this manuscript:

MDPI	Multidisciplinary Digital Publishing Institute
DOAJ	Directory of Open Access Journals
DJIA	Dow Jones Industrial Average
E	Singular Value Decomposition Entropy
VAR	Vector Autoregression
IRF	Impulse Response Function

## Appendix A. DJIA Index Components

**DOW Jones Industrial Average Components**	
Companies	Abbreviation
Alcoa Inc. Common Stock	AA
American Express Company	AXP
Boeing Company	BA
Bank of America Corporation	BAC
Caterpillar, Inc.	CAT
Cisco Systems, Inc.	CSCO
Chevron Corporation	CVX
E.I. du Pont de Nemours and Com	DD
Walt Disney Company (The)	DIS
General Electric Company	GE
Home Depot, Inc. (The)	HD
Hewlett-Packard Company	HPQ
International Business Machines	IBM
Intel Corporation	INTC
Johnson & Johnson	JNJ
JP Morgan Chase & Co.	JPM
Kraft Foods Inc.	KFT
Coca-Cola Company (The)	KO
McDonald’s Corporation	MCD
3M Company	MMM
Merck & Company, Inc.	MRK
Microsoft Corporation	MSFT
Pfizer, Inc.	PFE
Procter & Gamble Company (The)	PG
AT&T Inc.	T
The Travelers Companies, Inc.	TRV
United Technologies Corporation	UTX
Verizon Communications Inc.	VZ
Wal-Mart Stores, Inc.	WMT
Exxon Mobil Corporation	XOM

## Appendix C. Unit Root Tests

**Table A1:** Unit root tests.

Country	Variable–Index	Variable—Log-Difference
Industrial Production	−2.168465	−4.495169 ***
CPI	−0.301688	−11.61486 ***
DOW Jones Industrial Index	0.472485	−17.24346 ***
Entropy—2 years	−2.127139	−17.64445 ***
Entropy—3 years	−1.871055	−18.98787 ***

Note: *** denotes statistical significance of the unit root test at 0.10 level.

## Appendix D. Lag Order Selection in the VAR Model

**Table A2:** Baseline model with federal funds rate.

Lag	LogL	LR	FPE	AIC	SC	HQ
0	−2458.668	-	11.03391	16.59036	16.65254	16.61525
1	−1587.786	1706.578	0.037061	10.89418	11.26728	11.04355
2	−1484.548	198.8275	0.021886	10.36733	11.05135 *	10.64117 *
3	−1451.704	62.14923	0.020768	10.31451	11.30945	10.71282
4	−1417.119	64.27979 *	0.019482 *	10.24996 *	11.55583	10.77274

Note: * indicates lag order selected by the criterion.

**Table A3:** Alternative model with shadow policy rate.

Lag	LogL	LR	FPE	AIC	SC	HQ
0	−2517.533	-	16.40145	16.98675	17.04894	17.01165
1	−1647.292	1705.321	0.055329	11.29489	11.66800	11.44426
2	−1557.577	172.7842	0.035789	10.85910	11.54313 *	11.13294 *
3	−1523.438	64.59972	0.033665	10.79756	11.79251	11.19587
4	−1494.506	53.77314 *	0.032807 *	10.77108 *	12.07695	11.29387

Note: * indicates lag order selected by the criterion.
